# Fecal Multidimensional Assay for Non-Invasive Detection of Colorectal Cancer: Fecal Immunochemical Test, Stool DNA Mutation, Methylation, and Intestinal Bacteria Analysis

**DOI:** 10.3389/fonc.2021.643136

**Published:** 2021-02-25

**Authors:** Shaobo Mo, Hui Wang, Lingyu Han, Wenqiang Xiang, Weixing Dai, Pengfei Zhao, Fengchun Pei, Zhixi Su, Chengcheng Ma, Qi Li, Zhimin Wang, Sanjun Cai, Hao Wang, Rui Liu, Guoxiang Cai

**Affiliations:** ^1^ Department of Colorectal Surgery, Fudan University Shanghai Cancer Center, Shanghai, China; ^2^ Department of Oncology, Shanghai Medical College, Fudan University, Shanghai, China; ^3^ Department of Research and Development, Singlera Genomics (Shanghai) Ltd., Shanghai, China; ^4^ Department of Medical Oncology and Cancer Institute, Shuguang Hospital, Shanghai University of Traditional Chinese Medicine, Shanghai, China; ^5^ Shanghai-MOST Key Laboratory of Health and Disease Genomics, Chinese National Human Genome Center, Shanghai Industrial Technology Institute, Shanghai, China; ^6^ Department of Cancer Institute, Fudan University Shanghai Cancer Center, Fudan University, Shanghai, China; ^7^ Department of Colorectal Surgery, Chang Hai Hospital, Naval Medical University, Shanghai, China

**Keywords:** colorectal cancer, fecal biomarker, methylation, human gut microbiome, cancer screening

## Abstract

**Background:**

Fecal immunochemical test (FIT), DNA mutation, DNA methylation, and microbial dysbiosis all showed promising in colorectal cancer (CRC) non-invasive detection. We assessed CRC detection with an assay combining all these strategies and investigated the effect of clinical features on the performance of this comprehensive test.

**Methods:**

We performed a multidimensional analysis study using stool samples collected from 108 patients with CRC, 18 patients with colorectal adenoma, and 36 individuals with no evidence of colorectal disease. The multidimensional analysis of stool samples including FIT, stool DNA (sDNA) tests for three methylated genes (Septin9, NDRG4, BMP3) and three mutated genes (KRAS, BRAF, PI3KCA) using next generation sequencing as well as detection of stool bacteria level of *Fusobacterium nucleatum* and *Parvimonas micra* using qPCR method. We used a linear support vector classification model to analyze the data.

**Results:**

The sensitivity of FIT alone was 69.4% for CRC and 11.1% for adenoma. Separately, the sensitivity of the detection of intestinal bacteria, DNA mutation, and DNA methylation for CRC was 58.3, 50.0, and 51.9%, respectively. The combination of FIT and sDNA tests had a sensitivity of 81.5% for CRC (AUC: 0.93, better than FIT alone, P = 0.017) and 27.8% for adenoma with 94.4% specificity. Sensitivity of the multidimensional test to detect CRC with stage II (84.6%) and III (91.9%) CRC was relatively higher (88.2%) than that of patients with stage I (60.0%) and stage IV (75.0%) (P = 0.024). The rate of CRC detection increased with tumor size (P = 0.008) and age (P = 0.04). Interestingly, the rate of CRC detection was higher in smoking persons than non-smokers with marginal significance (P = 0.08).

**Conclusions:**

The multidimensional assay of stool samples combining FIT and stool DNA tests further improved the diagnostic sensitivity for CRC. This could provide new approach for improvement of CRC screening and further demonstrations are warranted.

## Introduction

Colorectal cancer (CRC) is the third most common cancer with over 1.2 million new patients per year and the fourth leading cause of cancer-related death worldwide (1). The potential processes of colorectal carcinogenesis can be screened (2). Its incidence and mortality are steadily dwindling because of the application of programmatic screening, which has been demonstrated in numerous large, long-term follow-up studies. The Minnesota Colon Cancer Control Study showed a relative risk of 0.68 (95% CI: 0.56–0.82) among participants randomized to annual fecal occult blood test (FOBT) screening compared to the control group over 30 years of follow-up (3). The Nurses’ Health Study and the Nottingham trial also showed the use of colonoscopy/sigmoidoscopy and FOBT screening reduced colorectal cancer mortality (4). In addition, evidence supports and guidelines endorse several tests and strategies, and screening for colorectal cancer has been found to be cost-effective (5).

Despite the supporting evidence, recommendations, and availability of several screening tests, a large proportion of the U.S. population is not up to date with screening. For instance, screening compliance in the Nottingham trial was only around 60%, which signified that those tests still necessitate improving (4). Therefore, a simple, non-invasive test with high sensitivity may increase the compliance rate for patients with colorectal cancer and advanced precancerous lesions which thus could improve clinical outcomes.

More and more study revealed colorectal cancer arises from accumulated genetic and epigenetic alterations (6, 7). The microbial dysbiosis in human gut become a new study area of CRC development and progression (8, 9). But intestinal microecology still lack of researches to combine these strategies for non-invasive CRC detection. In this study, we evaluate a multidimensional stool analysis as a tool for colorectal cancer detection, the assays including fecal immunochemical test (FIT), DNA mutation, DNA methylation, and bacteria relative levels. The results showed multidimensional analysis greatly improved detection rate of colorectal cancer and promising for early screening.

## Methods

### Ethics Statement

The Ethical Committee and Institutional Review Board of the Fudan University Shanghai Cancer Center reviewed and approved this study protocol. All patients signed written informed consent.

### Study Design

In this study, we established a multidimensional analysis using stool samples for the detection of CRC or colorectal adenoma. Stool samples were collected before tumor removal of CRC or adenoma patients. The control stools were collected from control individuals with no evidence of colorectal disease. FIT was tested once the samples were received. Multiple stool DNA (sDNA) test was performed including three methylation markers (Septin9, NDRG4, and BMP3), three mutation genes (KRAS, BRAF, and PI3KCA), and two bacteria relative levels (*Fusobacterium nucleatum* and *Parvimonas micra*). To assess the performance of multidimensional stool analysis, stool samples were distributed in balanced to training and validation datasets. A linear support vector classification model was built based on the training set, and then the validation set was evaluated by the model with a pre-selected cut-off.

Cecum, ascending, hepatic angle, or transverse colon tumor were designated as right-sided tumor; splenic flexure, descending, sigmoid colon, and rectum were defined as left-sided tumor. And TNM stage was reclassified according to American Joint Committee on Cancer (AJCC) 8th edition (10). All CRC cases involved in our study were adenocarcinoma. As for adenomas, advanced adenomas were defined as the ones fulfilling any of these following criteria: villous or tubulovillous histologic features, size ≥10 mm or high-grade dysplasia (11).

### Stool Collection, Processing, and Storage

All stool samples were collected 7 days after diagnostic colonoscopy but before the removal of CRC or adenomas if there is any (12). Also, all patients were not on antibiotics or received any antibiotics within 4 weeks before stool collection. Some stools were collected to q-FOB sample collection tube according to manufacturer’s instruction. Remained stools were buffered with STE (500 mM Tris-HCl, 10 mM NaCl, 100 mM EDTA), and homogenized with a shaker device at final 1:4 (w/v) in STE. A 16 ml aliquot were used for DNA extraction. Homogenized stools were stored at −80℃ before DNA extraction.

### FIT

Before whole stool samples were buffed with STE, some stools were collected to q-FOB sample collection tube and tested with Fecal Occult Blood Gold Gel Stripe (W.H.P.M.INC) according to manufacturer’s instruction.

### Stool DNA Extraction

Stool DNA was extracted following the manufacturer’s instruction of E.Z.N.A. Stool DNA Kit (Omega). Humanized DNA was quantified using standard curve method. Stool DNA was diluted with 1,000 fold; 10, 1, 0.1, 0.01, 0.001 ng/µl NA12878 DNA was used to establish the standard curve. Primers targeted to hLINE-1 was used to quantification, the sequence of primers used is listed in **Table S1**.

### Mutation Assays

Ten ng humanized DNA was input for mutation assays, primers for target region amplify was in 4 μM final concentration. The 75 μl PCR mix was composed of 37.5 μl Phusion Blood Direct PCR Master Mix, 3 μl primer pool, and 34.5 μl sDNA. The thermal cycling comprised of 98°C 3 min, following 25 cycles of 98°C 15 s, 62°C 30s, and 72°C 30s, final extension at 72°C 3 min. PCR product was purified by 75 μl AMPure XP Beads and elute with 30 μl Low TE. Second PCR was performed with 12.5 μl Phusion Blood Direct PCR Master Mix, 6.25 μl nuclease free water, 0.75 μl DMSO, 2.5 μl index primers (5 μM), and 3 μl first PCR elute. The thermal cycling comprised of 98°C 3 min, following eight cycles of 98°C 15 s, 55°C 30s, and 72°C 30s, final extension at 72°C 5 min. Then 25 μl nuclease free water was added to each well after PCR, then purified by 40 μl AMPure XP Beads and elute with 20 μl Low TE. The libraries were quantified and loaded to Illumina Miseq or Nextseq sequencer for sequencing. Target sequence (**Table S2**) was amplified by primers annealed with target regions. The sequence of primers for library preparation-mutation detection is seen in **Table S1**. The mutation rate over 0.1% was counted and used for downstream analysis.

### Methylation Assays

Ten ng humanized DNA was input for HpaII digestion, another 10 ng humanized DNA without HpaII digestion used as control. Digestion was treated in 37°C 3 h, then 80°C 20 min for enzyme inactivation. Digestion product was beads purified and elute with 20 μl low TE. Primers for target region amplify was in 4 μM final concentration, primers for KRAS region were used as reference. The 75 μl PCR mix was composed of 37.5 μl Phusion Blood Direct PCR Master Mix, 3 μl primer pool, 20 μl eluted DNA, 13.75 μl nuclease free water, and 0.75 μl DMSO. The thermal cycling comprised of 98°C 5 min, following 25 cycles of 98°C 15 s, 66°C 30s, and 72°C 30s, final extension at 72°C 3 min. PCR product was purified by 75 μl AMPure XP Beads and elute with 11 μl Low TE. Second PCR was performed with 12.5 μl Phusion Blood Direct PCR Master Mix, 0.75 μl DMSO, 2.5 μl index primers (5 μM), and 9.25 μl first PCR elute. The thermal cycling comprised of 98°C 3 min, following eight cycles of 98°C 15 s, 55°C 30s, and 72°C 30s, final extension at 72°C 5 min. Then 25 μl nuclease free water was added to each well after PCR, then purified by 40 μl AMPure XP Beads and elute with 20 μl Low TE. The libraries were quantified and loaded to Illumina Miseq or Nextseq sequencer for sequencing. The sequence of primers for library preparation-methylation detection is listed in **Table S1**.

### Bacteria Relative Level Assays

Diluted sDNA with nuclease free water to final 0.2 ng/μl, qPCR assays for *Fusobacterium nucleatum*, *Parvimonas micra*, and universal 16S were performed in parallel. The 20 μl PCR mix was composed of 10 μl KAPA SYBR FAST qPCR master mix, 5 μl diluted sDNA, 1 μl primers (final 400 nM each), and 4 μl nuclease free water. The thermal cycling comprised of 95°C 5 min, following 40 cycles of 95°C 15 s, 58°C 25s, and 72°C 30s with florescence take. *Fusobacterium nucleatum*, *Parvimonas micra* relative level was calculated with delta Ct method with universal 16S as reference. The sequence of primers for *Fusobacterium nucleatum*, *Parvimonas micra*, and universal 16S detection is listed in **Table S1**.

### Sequencing Data Analysis

Paired end reads were merged to single end reads by pear (0.9.6) with parameter “-j 4 -v 20 -t 30 -n 30” to recover high quality original DNA fragments. Adapter and primer sequences at the end of reads were trimmed by trim_galore (0.4.0). Reads from each sample were mapped to the reference sequence hg19 using Burrows-Wheeler Aligner (BWA-mem, v0.7.12) (Li and Durbin, 2009). Only the samples with greater than 50,000 total sequencing reads and 10,000 on-target reads were chosen for further analyses. GATK3.4.0 was applied to detect mutations (DePristo et al., 2011). Integrative Genomics Viewer (IGV v 2.2; Broad Institute, Cambridge, MA, USA) was used for visual inspection of the aligned reads. Normalized methylation value of each target region was calculated as follows:

$$Methylation=\frac{\frac{Digested\;read\;counts\;of\;target\;methylation-sensitive\;amplicon}{Undigested\;read\;counts\;of\;target\;methylation-sensitive\;amplicon}}{\frac{Digested\;read\;counts\;of\;the\;KRAS\;control\;amplicon}{Undigested\;read\;counts\;of\;the\;\;KRAS\;control\;amplicon}}$$

Samples were divided into training dataset and validation datasets. Features involved were: “Q-FIT, Age, *Fn* relative level, *Pm* relative level, Tumor load (KRAS),Tumor load (BRAF),Tumor load (PIK3CA), Tumor load (known markers), chr7:140453136:A:T, chr12:25398285:C:A, chr12:25398285:C:G, chr12:25398285:C:T, chr12:25398284:C:G, chr3:178936095:A:C, chr3:178952085:A:T, chr12:25398282:C:A, chr3:178952085:A:G, chr12:25398281:C:A, chr12:25398255:G:T, chr3:178936082:G:A, chr3:178936091:G:A, chr3:178936092:A:G, chr3:178936095:A:T, chr12:25398284:C:A, chr12:25398284:C:T, chr12:25398281:C:T, chr3:178936092:A:C, chr3:178952003:G:A, chr12:25398275:C:T, Septin9 methylation, NDRG4 methylation and BMP3 methylation.”

The values of each feature were scaled between 0 to 1 with MinMaxScaler. After scaling, linear support vector classification model was built with the training dataset (sklearn 0.22.1). Putting specificity prior to sensitivity, we set the threshold as the maximum value of prediction value of normal samples minus a margin of 0.005 in training dataset.

## Result

### Clinicopathological Features

The study comprised 162 samples from 108 patients with CRCs, 18 patients with colorectal adenoma, and 36 healthy control with no evidence of colorectal disease (NED). The median age was 58 years (IQR: 26–86) and 58% (n = 94) were male. Respectively, 80 samples were randomly selected and used for training and the other 82 samples were used for validation. Detailed clinicopathological characteristics in the training and validation sets were listed in **Table 1**.

**Table 1:** Detailed clinicopathological characteristics of individuals in the training and validation sets.

	Training set	Test set	Combined	P
Age, median (range)	58 (26~86)	58 (26~79)	58 (26~86)	0.88
Sex, % man	65.0%	51.2%	58.0%	0.106
Control (NED)	16	20	36	0.3269
Adenoma				
n	7	11	18	
size, cm, median (range)	2.5 (1.5~4)	1 (0.6~3)	1.5 (0.6~4)	0.076
advanced adenomas(n)	5	7	12	0.12
CRC				
n	57	51	108	
size, cm, median (range)	4 (1~11)	4 (1.5~10)	4 (1~11)	0.487
stage, I/II/III/IV	12/23/16/6	8/16/21/6	20/39/37/12	0.51
Left/right-sided	39/18	35/16	74/34	0.76

NED, no evidence of disease; CRC, colorectal cancer.

### Detection Results of Mutations in Stool Samples

**Figure 1:**
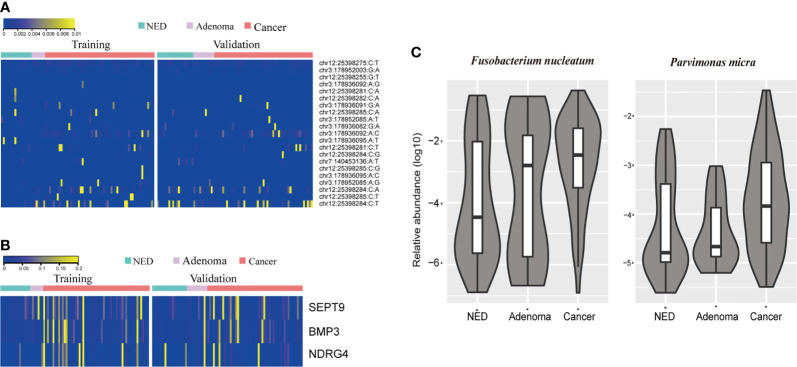
CRC patients accumulated more stool DNA mutation **(A)** and methylation **(B)** than adenoma and NED individuals. Quantitative PCR abundance of two bacteria markers of stool DNA showed relative higher level in adenoma and CRC patients’ samples than NED **(C)**.

The stool samples from CRC patients accumulated more mutations than adenoma and NED groups (**Figure 1A**). The results were consistent in training and validation data sets. Same as mutation markers, relative methylation level of Septin9, NDRG4, and BMP3 in CRC samples were higher than adenoma and NED groups (**Figure S1**), consistently in training and validation data sets (**Figure 1B**). The relative level of both *Fusobacterium nucleatum* and *Parvimonas micra* increased from NED, adenoma to CRC groups (**Figure 1C**).

Feature importance from the machine learning model was shown in **Figure S1**, which indicated that the weight of different features in the detection of CRC/adenoma varied. Therefore, to develop a multidimensional diagnosis model, we divided the samples to training and validation data sets in balanced group.

### Diagnostic Efficacy of Stool Samples

**Figure 2:**
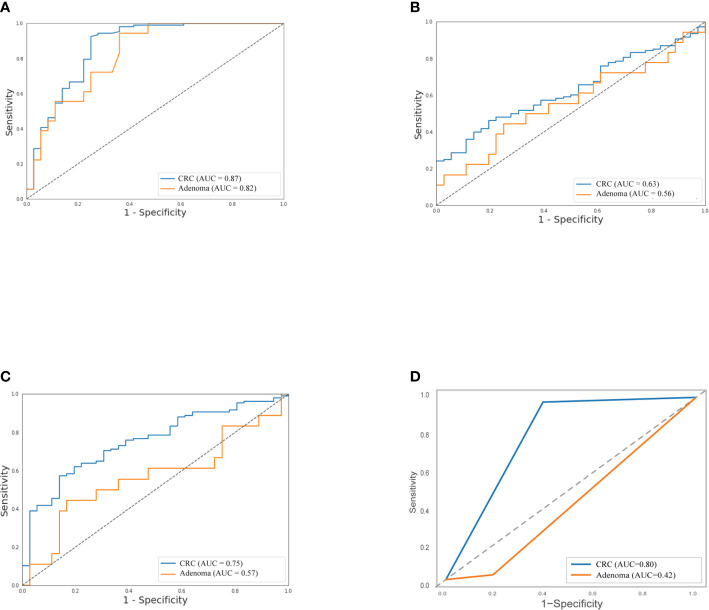
Separately, performance of stool DNA mutation **(A)** and FIT **(D)** to detect CRC were better than DNA methylation **(B)** and bacteria markers **(C)**, and stool DNA mutation predicted adenoma much precisely than the left three. The relative receiver operating characteristic (ROC) curves were shown.

As shown in **Table 2**, the sensitivity of FIT (69.4%) for CRC was higher than bacteria assay (58.3%), three gene mutations (50.0%), and DNA methylation (51.9%). And for adenoma, the sensitivities of FIT, bacteria assay, DNA mutation, and DNA methylation were 11.1, 38.9, 50.0, and 44.4%, separately. Generally, the specificity of FIT was highest (100%), while specificities of DNA mutation, DNA methylation, and bacteria assay were 88.9, 83.3, and 66.7% (**Table 2**). As ROC curves shown in **Figure 2**, FIT and genetic mutation were more accurate for predicting CRC than DNA methylation and bacteria markers. Also, DNA methylation performed better than bacteria markers in CRC screening.

**Table 2:** Diagnostic efficacy of separate bacteria assay, DNA mutation, DNA methylation, and FIT analysis.

	Bacteria assay	DNA mutation	DNA methylation	FIT
CRC sensitivity	58.3%	50.0%	51.9%	69.4%
Adenoma sensitivity	38.9%	50.0%	44.4%	11.1%
Specificity	83.3%	88.9%	66.7%	100%

CRC, colorectal cancer; FIT, fecal immunochemical test.

**Figure 3:**
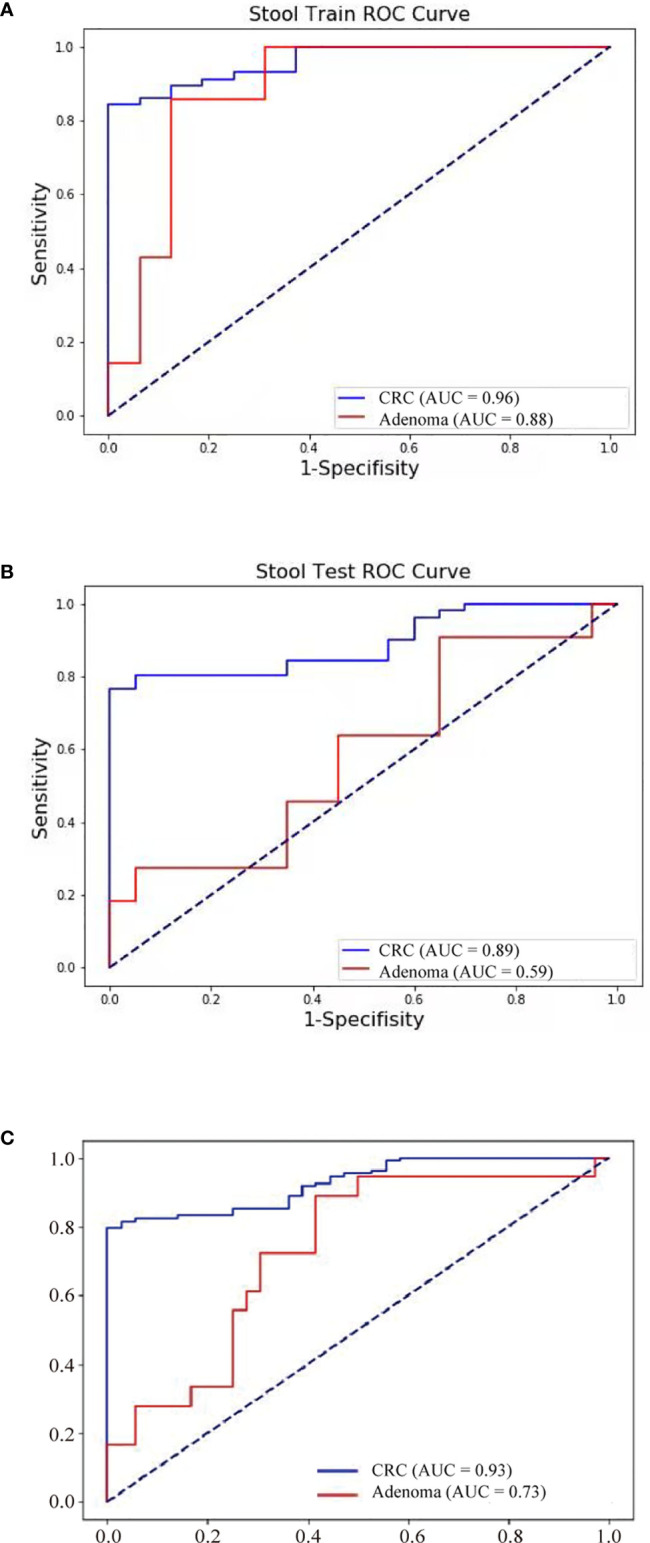
Neoplasm detection performance by multidimensional assay of stool samples. The sensitivity and specificity of training and validation datasets were consistent. ROC curves of the multidimensional assay in training set **(A)**, test set **(B)**, and combined data **(C)** were shown.

Further, FIT, methylation of three genes (Septin9, NDRG4, and BMP3), mutations in four regions of three genes (KRAS, BRAF, and PI3KCA), and bacteria relative levels of *Fusobacterium nucleatum* and *Parvimonas micra* were integrated to build a linear support vector model. Putting specificity prior to sensitivity, we set the threshold as the maximum value of prediction value of normal samples minus a margin of 0.005 in training dataset. The specificity of training data set was 93.8% with 84.2% cancer detection rate and 28.6% of adenomas. The performance of validation data set showed similar to training set, the specificity is 95%, CRC detection rate was 78.4%, and adenoma detection rate was 27.3%. The combined specificity was 94.4%, and combined sensitivity of CRC was 81.5% and 27.8% for adenoma (**Table 3**). Areas under the ROC curve were 0.93 for CRC and 0.73 for adenoma (**Figure 3**), which was better than FIT alone (AUC = 0.80) (P = 0.017).

**Table 3:** Neoplasm detection performance by multidimensional assay of stool samples.

	Training set	Test set	Combined
Sensitivity (CRC)	84.20%	78.40%	81.50%
Sensitivity (adenoma)	28.60%	27.30%	27.80%
Specificity	93.80%	95.00%	94.40%

CRC, colorectal cancer.

As for the influence of covariates on sensitivity, combined datasets were used for covariates analysis. The combination of FIT, sDNA tests, and bacteria level was significantly more sensitive for patient over 60 (90.38 *vs* 73%, P = 0.04). Also, the CRC detection rate increased with the tumor size (P = 0.008) (**Figure S2**). The CRC detection rate also increased from stage I (60.0%) to stage III (91.9%) while fell to 75.0% in stage IV patients. Sensitivity of CRC detection didn’t show difference with lesion location. Interestingly, there was a trend (P = 0.08) that the rate of CRC detection was higher in smoking persons than non-smokers (**Table 4**).

**Table 4:** The sensitivity of colorectal cancer (CRC) detection of stool multidimensional assay for the stratification by clinicopathological features.

Characteristics		N	Sensitivity (CRC)	P
Age	≤60	41/56	73%	0.04
	>60	47/52	90.38%	
Sex	Male	53/62	85.48%	0.32
	Female	35/46	76.09%	
Site	Left	60/74	81.08%	1.00
	Right	28/34	82.35%	
CRC stage	Stage I	12/20	60.00%	0.024
	Stage II	33/39	84.62%	
	Stage III	34/37	91.89%	
	Stage IV	9/12	75.00%	
CRC size (cm)	≤1	1/1	50.0%	0.008
	(1,2]	7/12	58.3%	
	(2,3]	13/18	72.2%	
	(3,4]	22/28	78.6%	
	>4	45/48	93.8%	
Smoker	Yes	29/31	93.5%	0.08
	No	59/77	76.6%	

CRC, colorectal cancer.

## Discussion

In our study, combination of FIT and sDNA was addressed for potential role in detection of CRC and adenoma, with 94.4% combined specificity. Our result indicated that this multidimensional stool model consisting of FIT, three methylation markers, three mutation genes, and two bacteria relative levels reached 94.4% specificity and 81.5% sensitivity of CRC. So far, our study is the first study on combined multidimensional sDNA assay including fecal DNA mutation, DNA methylation, and bacterial in Chinese population, with relative sensitivity of 81.5% for CRC and specificity of 94.4%.

In our cohort, the sensitivity of the synthetical model for CRC was nearly three times to adenomas; it performed better in stage III CRC and larger tumors. According to previous studies, the sensitivity of FIT varied from 30 to 73.8% (13, 14), which was closely related with TNM stage, and multiple molecular stool tests were demonstrated to outperform FIT. As diagnostic biomarker mentioned in previous study (15), methylated Septin9 (mSeptin9) from plasma alone achieved overall sensitivity of 61.8% (53.0–69.9%). An sDNA test approved by Food and Drug Administration (FDA), containing multiple DNA test (KRAS mutations and NDRG4 and BMP3 hypermethylation) and fecal hemoglobin, was validated with 92% sensitivity of CRC. However, this assay was largely limited in white population (16). Similar screening was conducted in Korean population, and the methylated NDRG4 and BMP3 was detected only in 68.8 and 40.0% of CRC, respectively (17). On the other hand, due to changes of gut microecology in incidence of CRC, other studies based on two *Fusobacterium species*, *Porphyromonas asaccharolytica*, and *Peptostreptococcus stomatis* explored the suitability of intestinal microbiota in CRC detection. Nevertheless, the ROC of these metagenomic classifiers were between 0.73 and 0.84 (18). Comparatively, the ROC of our new multidimensional assay for CRC reached 0.93.

This multiple sDNA test had better performance in stage II–IV, especially in stage III, which could be ideal supplement for FIT, consistent with the study by Li et al. showing highest sensitivity for stage III (68%) (15). Given the fact that the methylation markers and mutation genes are broadly expressed in CRC and adenoma, these molecules would be released into stool during cancer progression and vascular invasion. This explanation was also verified by the study showing that mSEPT9 test of peripheral blood samples presented highest sensitivity for stage III (84.1%) and stage IV (100%) (19). Decreased sensitivity in stage IV CRC in our study was due to limited sample and the fact that these tumors were smaller than average (median: 3.5cm, IQR: 3–6).

Age was demonstrated as clinical characteristic related to the sensitivity of multidimensional set in our study (P < 0.05). Defined as presence of methyl groups at CpG dinucleotides, DNA methylation was increasing with age. Further study illustrated that a small number of these certain CpG sites were highly associated with age, which even could be used for predicting age (20). Also, according to National Colorectal Cancer Screening Programme data, age was closely related to increasing sensitivity of FIT (21).

Notably, the rate of CRC detection was significantly higher in smoking persons than non-smokers in our study, which evidenced the advantage of this multidimensional test for colorectal tumor screening in smoking population. This may due to lifestyle changes (22) and immunosuppressive effect of cigarette smoking (23). Smoking CRC patients were found to be more likely to have a high CpG island methylator phenotype, indicating they had a higher level of multiple genes hypermethylation (24), which may explain the reason the above founding in our study. However, due to limited cohort of our study, this trend did not show statistical significance, which required further verification.

In line with knowledge that the low incidence of CRC in cancer screening, specificity is another important indicator for evaluating screening tools and reduces burden of screening follow-up colonoscopy for participants. Up to now, the specificity of sDNA in Chinese population was varied from 87 to 98% (25, 26). In the current study, the specificity was 94.4%, comparable to that in previous reports.

Also, there are several limitations in our study. First, as a retrospective study on cancer screening, our study only included colorectal cancer patients and healthy individuals, which did not include other colorectal non-neoplastic diseases such as ulcerative colitis and other gastrointestinal cancers such as gastric cancer. Moreover, there have been commercial stool DNA detection methods that have been used in clinical practice. However, head-to-head comparison with such commercial multitarget DNA test in feces (Cologuard™) was lack for screening for CRC (27). FIT test alone needs a small amount of stool, but patients had to collect more stools for our multidimensional assay and may feel inconvenient. Our multidimensional assay was also demanding in terms of technique and increased the cost of the screening. There was no external validation and the sample size was not large enough to establish a robust multidimensional assay since the assay was expensive and the budget was limited. A prospective, multicenter, large-scale trial was warranted to further certify the value of this assay since it has been shown to be promising in this preliminary study.

## Conclusion

The multidimensional assay of stool samples combining FIT and stool DNA tests further improved the diagnostic sensitivity for CRC. This preliminary study could provide a new approach for improvement of CRC screening. Further demonstrations on a large-scale study especially including more healthy population are warranted.

## Data Availability Statement

The original contributions presented in the study are included in the article/**Supplementary Material**. Further inquiries can be directed to the corresponding authors.

## Ethics Statement

The studies involving human participants were reviewed and approved by The Ethical Committee and Institutional Review Board of the Fudan University Shanghai Cancer Center. The patients/participants provided their written informed consent to participate in this study.

## Author Contributions

SC, HaoW, SM, HuiW, RL, and GC had the idea for this study. SM, WX, WD, and LH collected specimens. PZ, FP, and HuiW performed the experiments. SC and GC supervised the acquisition of the data. ZS and CM performed NGS data analysis. SM and HuiW undertook the statistical analysis. SC and GC provided statistical advice. All authors contributed to interpretation of the results. SM, LH and HuiW wrote the article. GC, SC, HaoW, and RL revised the article and other authors contributed to the content. All authors contributed to the article and approved the submitted version.

## Funding

This study was supported by the Grant of Science and Technology Commission of Shanghai Municipality (No. 17411951100) and the National Key Research and Development Program of China (grant number:2019YFC1315800).

## Conflict of Interest

HuiW, PZ, FP, ZS, CM and RL were employed by company Singlera Genomics (Shanghai). 

The remaining authors declare that the research was conducted in the absence of any commercial or financial relationships that could be construed as a potential conflict of interest.

## Supplementary Material

Supplementary Figure 1 Both mutation markers **(A)** and methylation level **(B)** in CRC samples were higher than NED groups. Furthermore, machine learning model depicted relevant feature importance **(C)**.

Supplementary Figure 2 Tumor size was highly correlated with CRC. detection rate.

Supplementary Table 1 Primer sequences used in present study.

Supplementary Table 2 The following target regions of known mutations for colorectal cancer(CRC) from mycancergenome.org were analyzed.

## References

[1] Ferlay J Soerjomataram I Dikshit R Eser S Mathers C Rebelo M. Cancer incidence and mortality worldwide: sources, methods and major patterns in GLOBOCAN 2012. Int J Cancer (2015) 136(5):E359–86. 10.1002/ijc.29210 25220842

[2] Vogelstein B Papadopoulos N Velculescu VE Zhou S Diaz LA Jr. Kinzler KW. Cancer genome landscapes. Science (2013) 339(6127):1546–58. 10.1126/science.1235122 PMC3749880 23539594

[3] Mandel JS Bond JH Church TR Snover DC Bradley GM Schuman LM. Reducing mortality from colorectal cancer by screening for fecal occult blood. Minnesota Colon Cancer Control Study. N Engl J Med (1993) 328(19):1365–71. 10.1056/NEJM199305133281901 8474513

[4] Scholefield JH Moss SM Mangham CM Whynes DK Hardcastle JD. Nottingham trial of faecal occult blood testing for colorectal cancer: a 20-year follow-up. Gut (2012) 61(7):1036–40. 10.1136/gutjnl-2011-300774 22052062

[5] Ladabaum U Mannalithara A. Comparative Effectiveness and Cost Effectiveness of a Multitarget Stool DNA Test to Screen for Colorectal Neoplasia. Gastroenterology (2016) 151(3):427–39 e6. 10.1053/j.gastro.2016.06.003 27311556

[6] Nguyen LH Goel A Chung DC. Pathways of Colorectal Carcinogenesis. Gastroenterology (2020) 158(2):291–302. 10.1053/j.gastro.2019.08.059 31622622 PMC6981255

[7] Berger BM Ahlquist DA. Stool DNA screening for colorectal neoplasia: biological and technical basis for high detection rates. Pathology (2012) 44(2):80–8. 10.1097/PAT.0b013e3283502fdf 22198259

[8] Dai Z Zhang J Wu Q Chen J Liu J Wang L. The role of microbiota in the development of colorectal cancer. Int J Cancer (2019) 145(8):2032–41. 10.1002/ijc.32017 PMC6899977 30474116

[9] Tilg H Adolph TE Gerner RR Moschen AR. The Intestinal Microbiota in Colorectal Cancer. Cancer Cell (2018) 33(6):954–64. 10.1016/j.ccell.2018.03.004 29657127

[10] Amin MB Greene FL Edge SB Compton CC Gershenwald JE Brookland RK. The Eighth Edition AJCC Cancer Staging Manual: Continuing to build a bridge from a population-based to a more “personalized” approach to cancer staging. CA Cancer J Clin (2017) 67(2):93–9. 10.3322/caac.21388 28094848

[11] Provenzale D Jasperson K Ahnen DJ Aslanian H Bray T Cannon JA. Colorectal Cancer Screening, Version 1.2015. J Natl Compr Canc Netw (2015) 13(8):959–68; quiz 68. 10.6004/jnccn.2015.0116 26285241

[12] Ahlquist DA Zou H Domanico M Mahoney DW Yab TC Taylor WR. Next-generation stool DNA test accurately detects colorectal cancer and large adenomas. Gastroenterology (2012) 142(2):248–56; quiz e25-6. 10.1053/j.gastro.2011.10.031 22062357 PMC4017869

[13] Imperiale TF Ransohoff DF Itzkowitz SH Levin TR Lavin P Lidgard GP. Multitarget stool DNA testing for colorectal-cancer screening. N Engl J Med (2014) 370(14):1287–97. 10.1056/NEJMoa1311194 24645800

[14] Shin A Choi KS Jun JK Noh DK Suh M Jung KW. Validity of fecal occult blood test in the national cancer screening program, Korea. PloS One (2013) 8(11):e79292. 10.1371/journal.pone.0079292 24260189 PMC3832630

[15] Xie L Jiang X Li Q Sun Z Quan W Duan Y. Diagnostic Value of Methylated Septin9 for Colorectal Cancer Detection. Front Oncol (2018) 8:247. 10.3389/fonc.2018.00247 30013949 PMC6036110

[16] Carethers JM. Fecal DNA Testing for Colorectal Cancer Screening. Annu Rev Med (2020) 71:59–69. 10.1146/annurev-med-103018-123125 31451044

[17] Park SK Baek HL Yu J Kim JY Yang HJ Jung YS. Is methylation analysis of SFRP2, TFPI2, NDRG4, and BMP3 promoters suitable for colorectal cancer screening in the Korean population? Intest Res (2017) 15(4):495–501. 10.5217/ir.2017.15.4.495 29142517 PMC5683980

[18] Zeller G Tap J Voigt AY Sunagawa S Kultima JR Costea PI. Potential of fecal microbiota for early-stage detection of colorectal cancer. Mol Syst Biol (2014) 10:766. 10.15252/msb.20145645 25432777 PMC4299606

[19] Jin P Kang Q Wang X Yang L Yu Y Li N. Performance of a second-generation methylated SEPT9 test in detecting colorectal neoplasm. J Gastroenterol Hepatol (2015) 30(5):830–3. 10.1111/jgh.12855 25471329

[20] Jones MJ Goodman SJ Kobor MS. DNA methylation and healthy human aging. Aging Cell (2015) 14(6):924–32. 10.1111/acel.12349 PMC4693469 25913071

[21] Sung NY Jun JK Kim YN Jung I Park S Kim GR. Estimating age group-dependent sensitivity and mean sojourn time in colorectal cancer screening. J Med Screen (2019) 26(1):19–25. 10.1177/0969141318790775 30261804

[22] Stevens C Smith SG Vrinten C Waller J Beeken RJ. Lifestyle changes associated with participation in colorectal cancer screening: Prospective data from the English Longitudinal Study of Ageing. J Med Screen (2019) 26(2):84–91. 10.1177/0969141318803973 30336731 PMC6484824

[23] Shiels MS Katki HA Freedman ND Purdue MP Wentzensen N Trabert B. Cigarette smoking and variations in systemic immune and inflammation markers. J Natl Cancer Inst (2014) 106(11):dju294. 10.1093/jnci/dju294 PMC4200029 25274579

[24] Nishihara R Morikawa T Kuchiba A Lochhead P Yamauchi M Liao X. A prospective study of duration of smoking cessation and colorectal cancer risk by epigenetics-related tumor classification. Am J Epidemiol (2013) 178(1):84–100. 10.1093/aje/kws431 23788674 PMC3698990

[25] Su XL Wang YF Li SJ Zhang F Cui HW. High methylation of the SEPT9 gene in Chinese colorectal cancer patients. Genet Mol Res (2014) 13(2):2513–20. 10.4238/2014.January.17.5 24535900

[26] Song L Jia J Peng X Xiao W Li Y. The performance of the SEPT9 gene methylation assay and a comparison with other CRC screening tests: A meta-analysis. Sci Rep (2017) 7(1):3032. 10.1038/s41598-017-03321-8 28596563 PMC5465203

[27] Tepus A Yau TO. Non-Invasive Colorectal Cancer Screening: An Overview Gastrointest Tumors (2020) 7(3):62–):73. 10.1159/000507701 32903904 PMC7445682

