# Supplementary figures and images for: Fecal Multidimensional Assay for Non-Invasive Detection of Colorectal Cancer: Fecal Immunochemical Test, Stool DNA Mutation, Methylation, and Intestinal Bacteria Analysis
Source: Front Oncol. 2021 Feb 25;11:643136. doi: 10.3389/fonc.2021.643136 (PMC7947614; doi:10.3389/fonc.2021.643136)

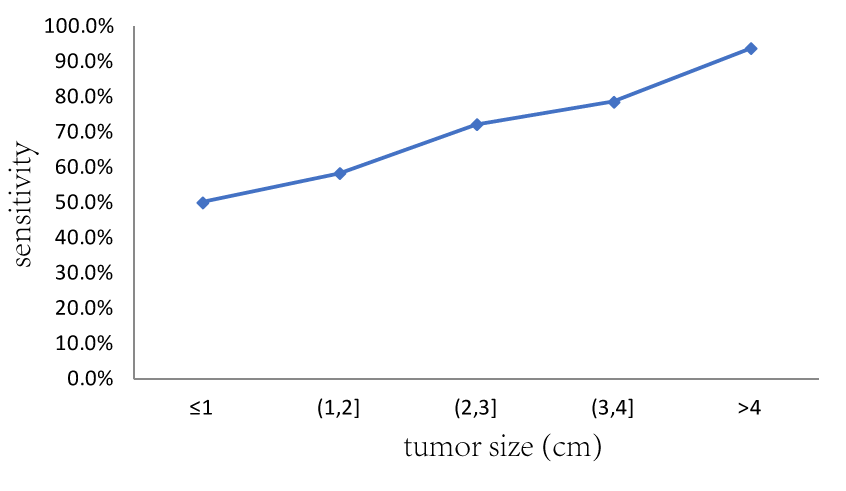

Supplement: Supplementary Figure 1 — Both mutation markers (A) and methylation level (B) in CRC samples were higher than NED groups. Furthermore, machine learning model depicted relevant feature importance (C). [file Image_1.tif]

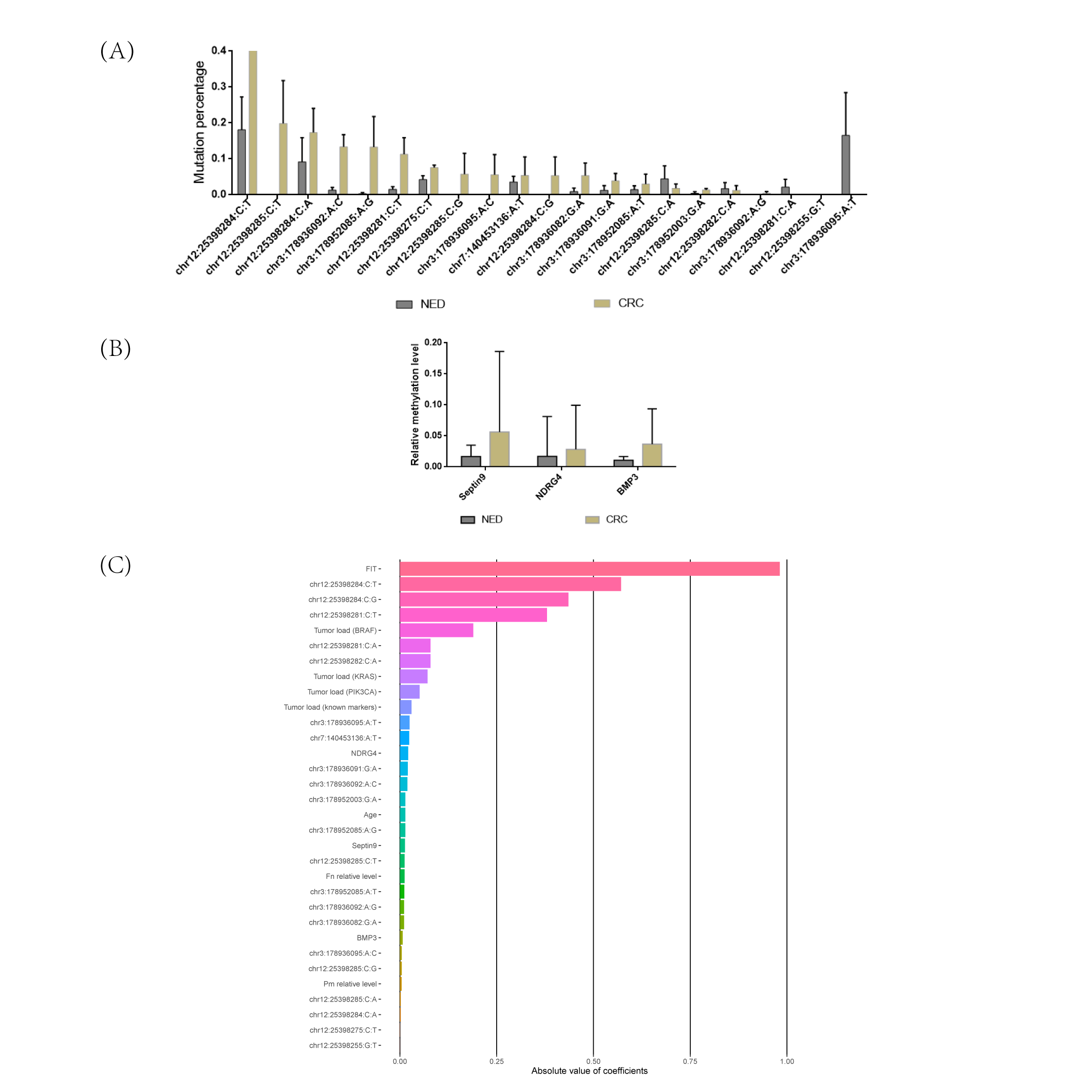

Supplement: Supplementary Figure 2 — Tumor size was highly correlated with CRC. detection rate. [file Image_2.tif]
