# Supplementary material for: Fecal Multidimensional Assay for Non-Invasive Detection of Colorectal Cancer: Fecal Immunochemical Test, Stool DNA Mutation, Methylation, and Intestinal Bacteria Analysis
Source: Front Oncol. 2021 Feb 25;11:643136. doi: 10.3389/fonc.2021.643136 (PMC7947614; doi:10.3389/fonc.2021.643136)
Supplement: Supplementary Table 1 — Primer sequences used in present study. [file DataSheet_1.docx]

Table S1. Primer sequences used in present study.

| qPCR Assays | Forward | Reverse |
| --- | --- | --- |
| hLINE-1 | TCACTCAAAGCCGCTCAACTAC | TCTGCCTTCATTTCGTTATGTACC |
| Fusobacterium nucleatum | GGATTTATTGGGCGTAAAGC | GGCATTCCTACAAATATCTACGAA |
| Parvimonas micra | AGAGTTTGATCCTGGCTCAG | ATATCATGCGATTCTGTGGTCTC |
| universal 16S | GGTGAATACGTTCCCGG | TACGGCTACCTTGTTACGACTT |
|  |  |  |
| library preparation-mutation | Forward | Reverse |
| KRAS | TCGTCGGCAGCGTCAGATGTGTATAAGAGACAGTACCTCTATTGTTGGATCATATTCGTCCA | GTCTCGTGGGCTCGGAGATGTGTATAAGAGACAGTATTATAAGGCCTGCTGAAAATGACTGAAT |
| BRAF | TCGTCGGCAGCGTCAGATGTGTATAAGAGACAGTCAGTGGAAAAATAGCCTCAATTCTTACC | GTCTCGTGGGCTCGGAGATGTGTATAAGAGACAGCTTCATGAAGACCTCACAGTAAAAATAGGT |
| PI3KCA | TCGTCGGCAGCGTCAGATGTGTATAAGAGACAGGCTAGAGACAATGAATTAAGGGAAAATGACA | GTCTCGTGGGCTCGGAGATGTGTATAAGAGACAGACAGAGAATCTCCATTTTAGCACTTACC |
| PI3KCA | TCGTCGGCAGCGTCAGATGTGTATAAGAGACAGTGGAATGCCAGAACTACAATCTTTTGAT | GTCTCGTGGGCTCGGAGATGTGTATAAGAGACAGGTGGAAGATCCAATCCATTTTTGTTGTC |
|  |  |  |
| library preparation-methylation | Forward | Reverse |
| Septin9 | TCGTCGGCAGCGTCAGATGTGTATAAGAGACAGTTCATTCAGCTGAGCCAGGG | GTCTCGTGGGCTCGGAGATGTGTATAAGAGACAGGTCCGACATGATGGCTGGTG |
| BMP3 | TCGTCGGCAGCGTCAGATGTGTATAAGAGACAGCTCGCCCCAGCTGGTTTG | GTCTCGTGGGCTCGGAGATGTGTATAAGAGACAGAAAGGTGGCTTCGGTCTCTC |
| NDRG4 | TCGTCGGCAGCGTCAGATGTGTATAAGAGACAGGGATCGACCGGGGTGTCC | GTCTCGTGGGCTCGGAGATGTGTATAAGAGACAGGTGGGTTTGGCGGGTGTG |
| qPCR Assays | Forward | Reverse |
| hLINE-1 | TCACTCAAAGCCGCTCAACTAC | TCTGCCTTCATTTCGTTATGTACC |
| *Fusobacterium nucleatum* | GGATTTATTGGGCGTAAAGC | GGCATTCCTACAAATATCTACGAA |
| *Parvimonas micra* | AGAGTTTGATCCTGGCTCAG | ATATCATGCGATTCTGTGGTCTC |
| universal 16S | GGTGAATACGTTCCCGG | TACGGCTACCTTGTTACGACTT |

| Table S2. The following target regions of known mutations for colorectal cancer(CRC) from *mycancergenome.org* were analyzed. | | | |
| --- | --- | --- | --- |
| Chromosome | Position | Reference | Alternate |
| chr7 | 140453136 | A | T |
| chr12 | 25398285 | C | A |
| chr12 | 25398285 | C | G |
| chr12 | 25398285 | C | T |
| chr12 | 25398284 | C | G |
| chr3 | 178936095 | A | C |
| chr3 | 178952085 | A | T |
| chr12 | 25398282 | C | A |
| chr3 | 178952085 | A | G |
| chr12 | 25398281 | C | A |
| chr12 | 25398255 | G | T |
| chr3 | 178936082 | G | A |
| chr3 | 178936091 | G | A |
| chr3 | 178936092 | A | G |
| chr3 | 178936095 | A | T |
| chr12 | 25398284 | C | A |
| chr12 | 25398284 | C | T |
| chr12 | 25398281 | C | T |
